# Compression Syndromes of the Stylohyoid Complex: Neural, Arterial, and Venous Phenotypes in a Dynamic Neurovascular Corridor

**DOI:** 10.3390/tomography12090127

**Published:** 2026-09-07

**Authors:** Diana Alexandra Bănică, Vladimir Ioan Zamfirescu, Mugurel Constantin Rusu, Iulian Brezean

**Affiliations:** 1 Division of Anatomy, Department 1, Faculty of Dentistry, “Carol Davila” University of Medicine and Pharmacy, 050474 Bucharest, Romania; diana-alexandra.banica2025@stud.umfcd.ro (D.A.B.); vladimir-ioan.zamfirescu2024@stud.umfcd.ro (V.I.Z.); 2 Discipline of General Surgery and Qualified Care in Surgical Specialties, Department of Specific Disciplines, Faculty of Midwifery and Nursing, “Carol Davila” University of Medicine and Pharmacy, 050474 Bucharest, Romania; iulian.brezean@umfcd.ro; 3 Department of General Surgery, “Dr. Ion Cantacuzino” Clinical Hospital, 030167 Bucharest, Romania

**Keywords:** Eagle syndrome, styloid process, stylohyoid complex, internal jugular vein, venous outflow obstruction, carotid artery dissection, styloidectomy

## Abstract

**Background/Objectives:** Compression syndromes of the stylohyoid complex (SHC) encompass neural, arterial, and venous presentations extending beyond classic Eagle syndrome. Symptomatic disease is increasingly recognised as a dynamic neurovascular conflict determined not by styloid process (SP) length alone, but by three-dimensional orientation, vessel and nerve proximity, and positional musculoskeletal interactions. This review synthesises that literature and proposes a phenotype-based framework integrating neural, arterial, venous, and mixed presentations. **Methods:** This is a narrative, pathophysiologically organised review rather than a systematic or scoping review. Landmark anatomical, clinical, and cohort studies were synthesised alongside systematic reviews, meta-analyses, osteological data, and selected case reports illustrating mechanisms not captured at cohort level. Evidence strength was interpreted by study design, distinguishing pooled estimates from retrospective series and proposed mechanisms. **Results:** The SHC and adjacent C1 transverse process form a narrow corridor containing the internal jugular vein, carotid arteries, and lower cranial nerves. SP elongation is common, but symptomatic compression depends more on orientation, SP-to-C1 distance, vessel proximity, and dynamic positional factors than on length. Three-dimensional CT angiography/venography is the anatomical cornerstone for defining morphology and vessel relationships, whereas catheter venography with manometry remains the haemodynamic reference standard in selected venous cases. Jugular narrowing is frequent in asymptomatic individuals and is not diagnostic without clinical and haemodynamic correlation. Surgical decompression appears favourable in selected patients, whereas primary endovascular stenting, evaluated only in single-centre observational series, carries a substantial complication profile and should generally be reserved for refractory or combined presentations. **Conclusions:** SHC compression syndromes are best understood as overlapping neural, arterial, venous, and mixed phenotypes within a dynamic neurovascular corridor. A phenotype-based classification and a cautious “styloidectomy-first” strategy for osseous venous compression are proposed as practical frameworks, but require prospective validation.

## Simple Summary

The styloid process is a thin spike of bone at the base of the skull. In some people it is longer or differently angled than usual, and it can press on the nerves, arteries, and veins that run beside it in the neck. Depending on which structure is squeezed, patients may have throat and face pain, stroke-like events, or headache and pressure from obstructed blood drainage from the head. This review brings these presentations together into a single framework based on which structure is compressed, and sets out what imaging is needed and which treatments are supported by current evidence.

## 1. Introduction

Documentation of SHC anomalies dates to Pietro Marchetti (1652), who described an elongated styloid process (SP) associated with stylohyoid ligament (SHL) ossification and respiratory symptoms [1,2,3,4,5]. Weinlechner (1872) published the first authenticated clinical description and performed the first documented surgical excision of an abnormally elongated SP [4,5,6]. Eagle’s systematic publications (1937–1958) defined “stylalgia” as a distinct clinical entity and identified SP elongation exceeding 25–30 mm—or complete stylohyoid ligament mineralization—as a cause of pharyngeal pain, otalgia, and headache [1,4,5,6,7,8,9,10,11]. Eagle distinguished two subtypes: the classic post-tonsillectomy form, driven by fibrous scar tissue irritating the glossopharyngeal nerve over the SP tip; and the stylo-carotid artery syndrome, arising from SP impingement on the internal or external carotid artery (ECA) and the periarterial sympathetic plexus [1,4,8,9,12,13]. Only a minority of individuals with stylohyoid-complex abnormality are symptomatic—on the order of 4% in Eagle’s own account, rising substantially when stylohyoid ligament calcification is included—which underlies the persistent gap between the high radiological prevalence of elongation and the rarity of clinical disease [14].

Subsequent radiological and surgical advances have broadened this concept to encompass a wider spectrum of neurovascular entrapment [13,15]. A venous phenotype—most accurately termed styloidogenic jugular venous compression syndrome (SJVCS), and historically labelled Eagle jugular syndrome or Styloid Jugular Nutcracker—has been described in which the J3 segment of the internal jugular vein (IJV)—the superior portion traversing the C1 osteomuscular corridor from the jugular foramen to the carotid bifurcation [16]—is compressed between the SP (or calcified SHL) and the C1 lateral mass [3,11,15,17,18,19,20,21]. This compression has been associated with retrograde venous hypertension, raised intracranial pressure, and, in selected severe cases, complications including cerebral venous sinus thrombosis. These associations are drawn principally from retrospective case series rather than prospective or population-based data, and the absolute frequency of severe complications among all individuals with anatomical compression remains poorly defined. The eponym “Eagle jugular syndrome” is increasingly regarded as a misnomer, since Eagle himself never described venous pathology; “styloidogenic jugular venous compression syndrome” is the more anatomically accurate designation and is used preferentially throughout this review, with alternative historical terms noted once and not reused [16].

Additional, non-styloid compression mechanisms have also been reported, including hypertrophic C1 lateral masses, anomalous omohyoid tendons, sternocleidomastoid (SCM) and omohyoid muscular entrapment [22], cervical spondylosis [17], transient rotational stenosis (“jugular bow hunter’s syndrome”) [23], and traumatic subcutaneous emphysema [24]. These non-styloid mechanisms are addressed in Section 4 because they frequently coexist with, confound, or mimic styloidogenic compression in clinical practice, even though they fall outside the SHC itself. This review (i) outlines the morphological determinants of compression, (ii) contrasts neural, arterial, and venous clinical phenotypes, (iii) evaluates the evidence for dynamic diagnostic modalities, and (iv) compares conservative, surgical, and endovascular management, while explicitly distinguishing well-established anatomical findings from emerging clinical associations and from the authors’ own proposed synthesis.

### Approach to the Literature

This review is narrative rather than systematic, and no pre-registered protocol or PRISMA-guided selection process was applied. PubMed/MEDLINE, Scopus and Web of Science were searched using combinations of the terms Eagle syndrome, elongated styloid process, stylohyoid complex, styloidogenic jugular venous compression, internal jugular vein stenosis and styloidectomy, supplemented by citation tracking of the reference lists of retrieved articles. Only English-language publications were considered. Retrieval spanned the literature from Eagle’s original description in 1948 to 2026, with approximately half of the cited sources published from 2020 onward, reflecting the recent expansion of interest in the venous phenotype. Selection was purposive rather than exhaustive: landmark anatomical and clinical studies, systematic reviews and meta-analyses, and large imaging or osteological cohorts were prioritised, with individual case reports included only where they document a mechanism not captured at cohort level. The review was prepared with reference to the SANRA criteria for narrative review articles [25], which informed the description of the search strategy, the explicit grading of evidence level, and the presentation of quantitative endpoint data. Because sources were not identified through a pre-specified search strategy, publication bias toward unusual or successfully treated presentations cannot be excluded (see Section 8).

## 2. Anatomy and Morphological Determinants

### 2.1. Embryology and Anatomy

**Figure 1:**
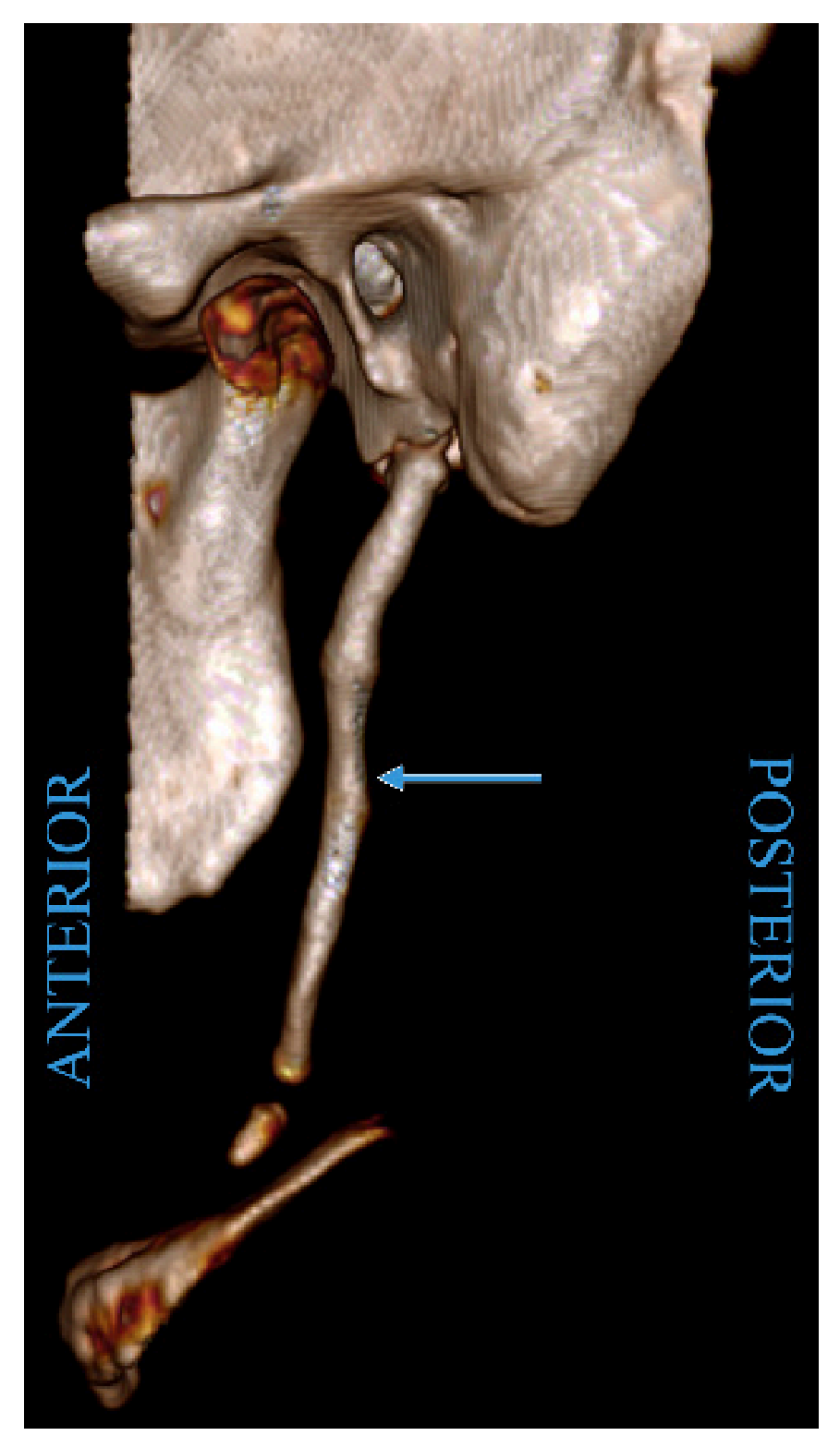
Elongated left styloid process (arrow). Left side, lateral view. Computed tomography. Three-dimensional volume rendering.

The styloid process (SP) is a slender, cylindrical projection of the petrous part of the temporal bone that projects anteroinferiorly from just anterior to the stylomastoid foramen towards the parapharyngeal space. In the adult, it typically measures 20–30 mm in length and tapers to a needle-like tip, and it serves as the attachment for three muscles (styloglossus, stylopharyngeus, and stylohyoid) and two ligaments (stylohyoid and stylomandibular) that make Riolan’s bouquet. Coursing between the internal and external carotid arteries and immediately lateral to the IJV, the SP lies in intimate relation to cranial nerves IX–XII and the cervical sympathetic trunk. This arrangement underlies its capacity to compress or irritate neural, arterial, and venous structures when elongated or abnormally angulated (Figure 1).

The SHC comprises the SP of the petrous temporal bone, the SHL, and the lesser horn of the hyoid bone [13,26], derived embryologically from Reichert’s cartilage (second pharyngeal arch) and traditionally subdivided into four segments: tympanohyal (SP base), stylohyal (SP shaft), ceratohyal (normally degenerates into the SHL), and hypohyal (lesser hyoid horn) [4,5,6,8,13,16,26]. The mature SP projects anteroinferiorly into the parapharyngeal space, anchoring Riolan’s bouquet, and passes between the internal and external carotid arteries adjacent to cranial nerves IX–XII and the sympathetic trunk [4,5,8,13]. At the C1 level, the J3 IJV traverses a rigid osteomuscular corridor bounded posteriorly by the C1 transverse process and anteriorly by the SP or ossified SHL [16,27], and a retrostyloid course of the ECA—reported in 11.88% of CT angiogram sides in one series—places that vessel at additional risk during surgical approaches to this region [28]. In endoscopic cadaveric dissections, the IJV has been found posteromedial to the SP in roughly two-thirds of sides and posterolateral in the remainder. Parapharyngeal tumours arising in the retrostyloid compartment can partially or completely compress and displace the vein, which carries practical implications for surgical planning in this space [29]. A CT-angiographic study of 120 heminecks quantified how these relationships shift with morphology: both the internal and external carotid arteries lay significantly closer to the styloid process when it was elongated, and the internal carotid was closer still when the stylohyoid chain was variably ossified, reinforcing that carotid proximity is governed by three-dimensional relationship rather than length alone. Three reproducible topographical patterns were defined at the styloid tip—ICA medial, ECA lateral, and IJV posterior in 80% of sides (the classical arrangement); both arteries anterior to the styloid in 14.2%; and both arteries posterior in 5.8%—providing an anatomical framework for anticipating which vessel is at risk in a given patient [30]. The absence of an SP is itself a variable entity: ‘true absence’ denotes aplasia of both tympanohyal and stylohyal segments, whereas ‘pseudo-absence’ reflects a hypomineralised tympanohyal masked by the vaginal process of the tympanic plate and is a recognised radiological pitfall [31].

### 2.2. Styloid Morphology and Prevalence

The typical adult SP measures 20–30 mm [6,8,13,32]. A systematic review and meta-analysis of 104 studies and 136,010 heminecks reported pooled prevalences of 74.97% for a typical SP and 25.03% for elongation (of at least 30 mm), with a pooled mean length of 28.91 ± 1.73 mm; this is the strongest available evidence on SP prevalence and is reported here as a pooled estimate accordingly [13]. Direct osteological measurement is broadly concordant with these imaging-derived pooled values: in a collection of 250 dried skulls, the mean styloid length was 26.57 mm (SD 7.40 mm), reinforcing that the upper limit of normal lies close to 30 mm across both radiological and cadaveric datasets [33]. A large multidetector-CT series of 805 patients (1610 stylohyoid chains) similarly reported a mean styloid length of 30.5 mm—significantly greater in males than females (33.2 vs. 29.6 mm). Elongation exceeded 30 mm in 56% of patients and peaked in the fifth decade. Catalogued structural variants included segmentation, duplication (1.6%), and stylohyoid ligament ossification (3.0%) [34]. The same systematic review reports pooled prevalences of 16.35% for Langlais Type I (continuous) ossification, 4.39% for Type II (pseudo-articulated), and 3.89% for Type III (segmented) [13,16]. By contrast, isolated osteological and case-level observations of extreme elongation or rare morphological variants are described once, in selected studies, and should not be read as population-representative [10,26]. Single-population imaging series remain consistent with this pooled picture: in a cross-sectional study of 400 panoramic radiographs from Barcelona, 72.75% showed an elongated styloid process (ESP) exceeding 30 mm, most commonly the uninterrupted Langlais Type I morphology, yet only approximately 9.6% of individuals with an ESP were symptomatic [35]. The apparent discordance between these figures—25.03% in pooled meta-analysis, 56% in the multidetector-CT series, and 72.75% in the panoramic cohort—reflects differences in diagnostic threshold, population, and measurement convention rather than modality. Panoramic and CT length measurements correlate closely (r = 0.92–0.97), and ESP prevalence determined by the two methods differs only marginally [36], so imaging technique alone does not account for the spread. The 30 mm cut-off is itself the principal source of variability: mean styloid length approaches or exceeds this value in several populations, so a threshold intended to define an outlier instead captures a large proportion of normal individuals, and prevalences of 30–60% have been reported consecutively across multiple cohorts [36]. Estimates should therefore be compared only within a given threshold definition and population, and the pooled meta-analytic figure treated as the most appropriate reference value.

Pathological compression is not determined by SP length alone [4,5,7,13,26]. Three-dimensional SP angulation and deviation, the SP-to-C1 distance, and the SP-to-vessel distance are reported as more discriminating morphological determinants of compression than length in isolation [6,16]. Steinman demonstrated that angular deviation alone—without absolute SP elongation—can produce classic Eagle symptoms [37]. The most controlled evidence for proximity as the primary geometric driver comes from a case–control CT study in which the SP-tip-to-tonsillar-fossa distance was 4.6 mm in symptomatic elongated-SP patients versus 12.0 mm in asymptomatic controls (*p* < 0.01). At the same time, SP length itself did not differ significantly between groups [38]. Age-related arteriosclerosis and disc degeneration are reported to convert congenital arterial curves into haemodynamically significant kinks over time [39], and dynamic interactions—head rotation, jaw movement, omohyoid contraction—may compound these effects independently of SP dimensions [19]. IJV calibre also decreases substantially in the upright position relative to supine [27,40].

The cellular mechanism underlying SP elongation—relevant to understanding why compressive symptoms often emerge progressively in middle age—has been characterised immunohistochemically: elongation represents an active osteogenetic process driven by mechanical traction at the tendinous insertions of the styloid musculature, with woven bone trabeculae deposited at sites of repetitive tensile stress [41,42]. Systemic conditions that alter mineral metabolism can accelerate this process: patients with end-stage renal failure exhibit significantly greater SP length and stylohyoid ligament mineralisation, attributable to abnormal calcium and phosphorus metabolism compounded by long-term dialysis [43,44,45]. An age-dependent progression is further supported by predictive modelling in which each additional year of age increased the probability of SP elongation by a factor of 1.05 [46]. These findings collectively suggest that symptomatic compression in the fourth to sixth decade of life may reflect cumulative, mechanically driven osteogenesis rather than a stable congenital variant.

### 2.3. Positional and Dynamic Determinants of Compression

The corridor bounded by the styloid process anteriorly and the C1 transverse process posteriorly is not a fixed aperture. Its cross-sectional dimensions, and the distance between each bony landmark and the vessels and nerves that traverse it, change continuously with head position, mandibular movement and the phase of deglutition. Because compression is a function of clearance rather than of styloid length in isolation, these physiological excursions are a determinant of symptom expression in their own right, and they explain why anatomically similar styloid processes may be asymptomatic in one individual and symptomatic in another.

Four movement patterns are relevant. Axial rotation is the most consequential: contralateral rotation narrows the styloid–C1 interval on the side towards which the vessel is displaced, and is the movement under which rotational jugular stenosis, sternocleidomastoid-mediated venous collapse, and the ‘self-stabbing’ mechanism of carotid injury have each been documented (Section 3.2, Section 3.3 and Section 4). Flexion and extension alter the vertical relationship between the styloid tip and the C1 transverse process and shift the point at which the vein is apposed to bone along its J3 segment. Mandibular movement and swallowing displace the styloid musculature and the tonsillar fossa relative to the styloid tip, which is the mechanism invoked for movement-provoked neural symptoms and for inferior alveolar nerve irritation (Section 3.1). Posture acts differently again: the supine-to-upright reduction in jugular calibre noted in Section 2.2 means that a corridor which is patent during recumbent imaging may not be patent when the patient is upright and symptomatic.

The diagnostic consequence is that a single neutral-position, supine study samples only one configuration of a corridor that varies throughout the day. This is the rationale for provocative-position imaging and for dynamic rotational venography, and it is the reason that anatomical narrowing on static imaging correlates poorly with symptoms in both directions—narrowing is frequent in asymptomatic individuals, while symptomatic positional compression may be invisible at rest (Section 5).

## 3. Clinical Phenotypes and a Proposed Classification Framework

### 3.1. Neural Phenotype

Classic Eagle syndrome occurs almost exclusively after tonsillectomy or pharyngeal trauma [1,4,5,8]. Periprocessual fibrous scar tissue is reported to tether the pharyngeal mucosa to the SP tip, generating tension across the glossopharyngeal and adjacent cranial nerves during swallowing or head rotation, resulting in unilateral pharyngeal pain, referred otalgia, dysphagia, globus pharyngeus, and trismus [1,4,5,7,8,9,26]. Contemporary pathophysiological models favour a nerve-entrapment mechanism over the historical hypothesis of reactive ossifying hyperplasia, though direct mechanistic confirmation in humans is limited [5,7]. The neural phenotype is not confined to the glossopharyngeal nerve: dynamic irritation of the inferior alveolar nerve by an elongated calcified styloid process during mandibular movement has been reported to produce progressive mental-nerve (numb chin) paraesthesia that resolved completely after styloidectomy, illustrating that mobile adjacent nerves beyond CN IX can be entrapped by the same mechanism [47].

### 3.2. Arterial (Stylo-Carotid) Phenotype

The stylo-carotid phenotype arises through direct SP impingement on the internal or external carotid artery and the periarterial sympathetic plexus, independently of tonsillectomy [1,4,5,7,8,9]. Medial SP deviation has been associated with internal carotid compression and parietal cephalalgia or Horner’s syndrome; lateral deviation has been associated with external carotid compression and orbital or facial pain [1,4,8,9,28]. Symptoms in this phenotype are characteristically provoked by head movement, consistent with a dynamic mechanical mechanism: a 49-year-old man with persistent cervical pain and a 7.5 cm styloid process was successfully treated by piezosurgical resection, with histology of the removed process supporting mechanical irritation of the carotid and its perivascular sympathetic fibres [48]. The morphometric risk for arterial injury follows a quantifiable dose–response pattern: each 5 mm increment in SP length was associated with an approximately twofold increase in the risk of cervical carotid artery dissection in a case–control CTA study [49]. A specific dynamic mechanism—the ‘self-stabbing phenomenon’—has been described in which forceful or sustained head rotation drives the SP tip directly into the internal carotid artery (ICA) wall, causing intimal injury, dissection, and potential thromboembolism at the moment of rotational stress [50]. A representative imaging-documented case illustrates this sequence: a 43-year-old man with bilateral elongated processes (37 mm) developed internal carotid dissection and occlusion, with transoral carotid ultrasonography demonstrating the double-lumen dissection flap and follow-up CT angiography confirming close apposition between the styloid tip and the recanalised artery [51]. The positional nature of this conflict is shown even more directly by a case of reversible left-hemispheric ischaemia that developed within 15 s of left head rotation and resolved fully on return to the neutral position, with surgical and CT-angiographic correlation confirming rotational styloid impingement on the carotid [52]. A literature review of 56 symptomatic vascular cases documented internal carotid dissection in 15 cases, thrombosis in 8, and severe stenosis in 2 [53]; these figures derive from pooled case-level reporting rather than a defined incidence cohort and should be interpreted as a description of reported case mix rather than a population risk estimate. Chronic mechanical injury has also been reported in association with extracranial carotid pseudoaneurysm formation, a rare but serious complication requiring surgical reconstruction [4,13,39,54]. A 2024 PRISMA-based review reinforced and qualified these observations: across 78 articles (68 case reports, 5 systematic reviews and 7 retrospective case–control studies), a link between stylohyoid-complex pathology and internal carotid dissection was supported, with a reduced SP-ICA distance emerging as the more consistent association (significant in 6 of 7 case–control studies) than SP length alone (4 of 7), and one study finding no association for length, distance or ligament calcification; recurrent stroke or TIA occurred in 29% of cases managed medically and 56% after ICA stenting without styloid resection, whereas no recurrence was reported after stylohyoid resection over a median 6-month follow-up [55]. The authors nonetheless graded the entire evidence base, including the systematic reviews, as methodologically weak, so these figures are best read as hypothesis-generating rather than as validated risk or outcome estimates [55]. This association has since been formalised quantitatively: a 2023 meta-analysis of four case–control studies (185 dissection cases, 278 controls) found the styloid process to be significantly longer on the dissection side, with the effect strengthening after removal of a single high-risk-of-bias study (mean difference 3.61 mm; ipsilateral-versus-contralateral difference 2.63 mm), and an SP longer than 30 mm associated with dissection on sensitivity analysis (pooled OR 2.09) [56]. The authors nonetheless cautioned that the pooled sample is small and the association not yet firmly established. Analogous bony conflict has also been attributed to the hyoid bone rather than the styloid process: a 2026 case report and systematic review compiled 25 cases of ischaemic stroke secondary to hyoid-carotid (or, more rarely, hyoid-vertebral) conflict published between 1999 and 2025, in which greater-horn proximity to the ICA precipitated dissection or embolism through the same repeated-microtrauma mechanism, reinforcing that the stylohyoid complex should be assessed in its entirety rather than by styloid length alone [57].

### 3.3. Venous Phenotype (SJVCS)

The venous phenotype is hypothesised to result from mechanical J3 IJV stenosis between the SP or calcified SHL and the C1 lateral mass [3,15,16,18,20,27]. Because the intracranial venous system has limited valvular competence, downstream obstruction may promote retrograde venous hypertension and impaired CSF resorption. However, the extent to which this occurs likely depends heavily on individual collateral venous capacity, and absolute statements about inevitability are not supported by current evidence [3,15,18,20,21,27,58]. A systematic literature review of 149 reported patients with SJVCS found a near-equal sex distribution, a mean reported onset age of 38.6 years, bilateral stenosis in 54.1%, and J3 segment involvement in 91.2% [21]. Reported presenting symptoms included headache (46.3%), pulsatile tinnitus (43.6%), and visual disturbance (28.9%), with objective intracranial pressure elevation recorded in 36.2% [2,15,20,21,27]. As this synthesis is drawn from heterogeneous published cases rather than a prospectively defined cohort, these proportions should be regarded as a description of the reported literature rather than population-level incidence. Severe complications, including cerebral venous sinus thrombosis and non-aneurysmal subarachnoid haemorrhage, have been described in selected cases, typically where collateral venous pathways were also compromised. However, their overall frequency among all patients with venous compression remains unestablished [15,18,20,21,58]. A de novo brainstem cavernous malformation attributed to chronic styloidogenic venous hypertension has been reported in a single case-based systematic review [59]; this is a correlational observation and should not be read as establishing causation.

### 3.4. A Proposed Classification Framework

To organise these phenotypes for clinical use, a four-category framework is proposed here and summarised in Table 1. This framework is the authors’ synthesis, intended as a working clinical tool rather than a validated or externally tested classification, and it is distinct from Eagle’s original two-subtype distinction. Earlier authors had already extended Eagle’s two subtypes into a four-syndrome scheme—classic Eagle syndrome, carotid artery syndrome, and the largely asymptomatic stylohyoid and pseudostylohyoid syndromes—linked, respectively, to reactive hyperplasia, metaplasia, and ageing-related developmental theories of ossification [60]; the present framework differs in being organised by the compressed neurovascular structure rather than by the presumed ossification mechanism. Within the arterial phenotype, subtypes IIa and IIb are not intended as a severity gradient but as distinct clinical risk profiles: IIa captures SP-mediated adventitial and periarterial sympathetic irritation with predominantly local or low-grade ischaemic features, whereas IIb designates cases in which arterial wall integrity is already compromised—with dissection, intramural thrombus, or critical stenosis—conferring substantially higher thromboembolic risk and typically mandating more urgent or staged management; a single patient may progress from IIa to IIb over time. Phenotypes frequently overlap in practice: bilateral SP elongation has been reported in 63% of vascular Eagle syndrome cases [53], and a connective tissue disorder diagnosis—most often Ehlers–Danlos syndrome—was present in a substantial proportion of one large venous outflow case series, though this figure is specific to that retrospective single-centre cohort and should not be generalised without further study [27]. The concurrent occurrence of Eagle-related extracranial carotid pseudoaneurysm and IJV nutcracker syndrome—reported as the EXTINCT syndrome (Stylohyoid Eagle syndrome and EXTracranial INternal Carotid arTery pseudoaneurysm combined with venous compression)—represents the most fully documented example of the Type IV mixed phenotype [54]. In Eastern cohorts, previous hepatitis B virus (HBV) infection and type 2 diabetes mellitus have been reported as the leading systemic associations with non-compressive IJV stenosis (IJVS); these associations are hypothesised to reflect chronic immunopathic vasculitis, are based on a specific Chinese clinical cohort in which selection and referral patterns cannot be separated from any true population difference, and require validation before being generalised beyond that population [61].

## 4. Non-Styloid Contributors to Internal Jugular Vein Compression

**Figure 2:**
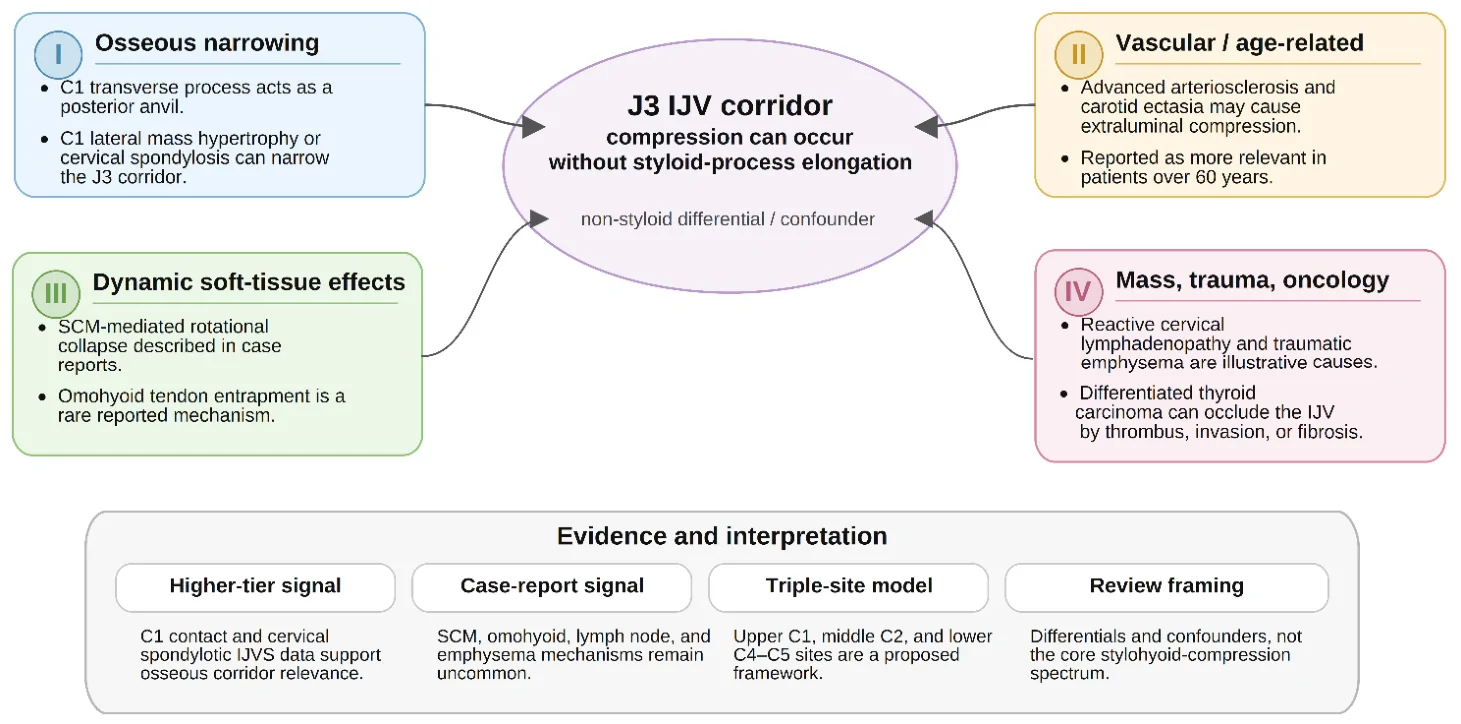
Non-styloid anatomical contributors to internal jugular vein (IJV) compression at the J3 corridor. Four categories of extrinsic compressors are illustrated. (**I**) Osseous narrowing: the C1 transverse process acts as a fixed posterior anvil; C1 lateral mass hypertrophy and cervical spondylosis can narrow the corridor independently of the length of the styloid process (SP). (**II**) Vascular and age-related mechanisms: advanced arteriosclerosis and carotid ectasia may cause extraluminal compression, particularly in patients over 60 years of age. (**III**) Dynamic soft-tissue effects: sternocleidomastoid (SCM)-mediated rotational IJV collapse and omohyoid tendon entrapment are described mechanisms, supported only by case-level evidence. (**IV**) Mass-, trauma-, and oncology-related causes: reactive cervical lymphadenopathy, traumatic emphysema, and differentiated thyroid carcinoma (via thrombus, direct invasion, or post-surgical fibrosis) have each been reported in isolated cases. The evidence-and-interpretation panel distinguishes higher-tier osseous evidence from case-report-level soft-tissue mechanisms. It situates the proposed triple-site conceptual model (upper C1, middle C2, lower C4–C5) within this framework. All four categories are presented as confounders and differential diagnoses of styloidogenic compression rather than as core components of the stylohyoid complex compression spectrum. IJV, internal jugular vein; SCM, sternocleidomastoid; SP, styloid process; IJVS, internal jugular vein stenosis; SHC, stylohyoid complex.

As outlined in Section 2.3, the C1 transverse process can act as a posterior anvil against which the J3 IJV is compressed independently of SP length, and hypertrophy of the C1 lateral mass or cervical spondylosis has been reported to narrow this corridor in its own right [16,17,18,27] (Figure 2). In a cadaveric study of 36 IJVs, the posterior vein wall was found in contact with the C1 transverse process in all specimens, with severe kinking reported in 8.3% [58]. In a prospective series of 46 patients with cervical spondylotic IJV stenosis (92 sides, 69 stenotic veins), compression was attributed to the C1 transverse process alone in 50.7% of stenotic vessels and to the C1 transverse process combined with the styloid process in 44.9%, with the J3 segment involved in 98.6%; notably, isolated styloidogenic cases were excluded from that cohort by design [17]. These figures derive from defined institutional case series and meta-analytic samples, respectively, and are reported at a higher evidentiary tier than the muscular and traumatic mechanisms below.

SCM-mediated rotational IJV collapse has been documented in case reports, including one in which the cross-sectional area reportedly fell by approximately 90% during head rotation [23]; this remains a described mechanism rather than an established population-level phenomenon. Similarly, omohyoid intermediate tendon entrapment, reactive cervical lymphadenopathy, and traumatic subcutaneous emphysema have each been described as causes of IJV compression in isolated or small case series and should be read as illustrative of possible mechanisms rather than as common causes [19,24,62,63,64]. Advanced arteriosclerosis and carotid ectasia have been reported to account for a meaningful proportion of extraluminal compression in one Chinese IJVS cohort, particularly in patients over 60 years of age [61]. A “triple compression site” model—upper (C1), middle (C2, beneath the digastric), and lower (C4–C5, near the SCM and carotid)—has been proposed to explain why treating only the styloidogenic site sometimes fails to restore venous flow fully [16]; this model is a conceptual framework rather than a validated diagnostic algorithm. A further anatomical variant relevant to this region is a long extracranial extension of the inferior petrosal sinus running parallel to the IJV before draining into it well below the skull base, sometimes termed an accessory IJV; this variant has been documented angiographically and on multi-slice CT, with reported extracranial lengths of up to approximately 100 mm [65,66]. In oncological settings, differentiated thyroid carcinoma is a separately characterised cause of IJV occlusion via thrombosis, tumour thrombus, direct invasion, or post-surgical fibrotic collapse. It is mechanistically distinct from styloidogenic or musculoskeletal compression [67].

Congenital craniovertebral junction anomalies, such as unilateral C1 lateral mass hypertrophy, represent a further rare non-styloid contributor to bony crowding in this region. However, these have been reported primarily as a cause of spinal cord compression and myelopathy rather than isolated venous obstruction [68,69].

Because these non-styloid mechanisms fall outside the stylohyoid complex itself, they are presented here as relevant differential and confounding factors rather than as part of the core stylohyoid-compression spectrum that is the principal focus of this review. An additional finding of structural co-occurrence relevance is the ponticulus posticus—a bony arch bridging the vertebral artery groove on the posterior arch of the atlas—which has been reported to occur significantly more frequently in individuals with ESPs, suggesting that bony developmental overcrowding may affect multiple sites in the same individual [70].

## 5. Clinical Presentation, Diagnosis, and Differential Diagnosis

Neural-phenotype symptoms reported across case series include globus sensation, pharyngeal pain, cervicalgia, dysphagia, and referred otalgia [1,4,5,6]. Arterial-phenotype presentations include carotidynia, pulsatile bruit, parietal headache, syncope, and TIA, with visual deficit, hemiparesis, dysarthria, and Horner’s syndrome reported in varying proportions across pooled case series [4,6,8,39]. Venous-phenotype presentations are dominated by sleep disturbance, head noise, tinnitus, dizziness, and headache [16], and a small series of patients with SCM/omohyoid entrapment additionally reported shoulder pain, arm numbness, and cognitive slowing [22]; papilledema and macular oedema have been described in severe cases but are not universal features [20,27,40]. Diagnostic delay across all phenotypes has been estimated at over three years in pooled case-series data, reflecting the non-specific nature of these presentations [6].

Diagnostic modalities for stylohyoid-complex compression, together with their principal contribution and key limitation, are summarised in Table 2. Three-dimensional CT angiography/venography (3D-CTA/CTV) with multiplanar reconstruction is the anatomical cornerstone for evaluating SP morphology, orientation, and proximity to adjacent vessels [4,12,13,16,18,20,28,71]. However, moderate-to-severe IJV narrowing has been reported incidentally in a substantial proportion of asymptomatic individuals undergoing CT angiography [27,72], so anatomical narrowing on static imaging is not in itself diagnostic of a symptomatic outflow disorder; imaging must be obtained in both neutral and provocative positions and interpreted alongside clinical correlation. Quantitative Doppler ultrasound and MR venography/angiography provide complementary functional and structural information [2,11,16,19,20,22,71], while catheter venography with retrograde manometry is the haemodynamic reference standard for confirming a clinically meaningful trans-stenotic pressure gradient in selected symptomatic venous cases [18,21,22,27]. In a descriptive study of 89 patients undergoing dynamic rotational venography, 93.3% developed at least moderate positional stenosis in at least one head position, and a clinically meaningful subset exceeded the 8 mmHg trans-stenotic pressure gradient threshold commonly used to define haemodynamically significant stenosis; these findings come from a single-centre descriptive series and should be interpreted as hypothesis-generating regarding the prevalence of dynamic compression rather than as definitive population estimates [40].

**Figure 3:**
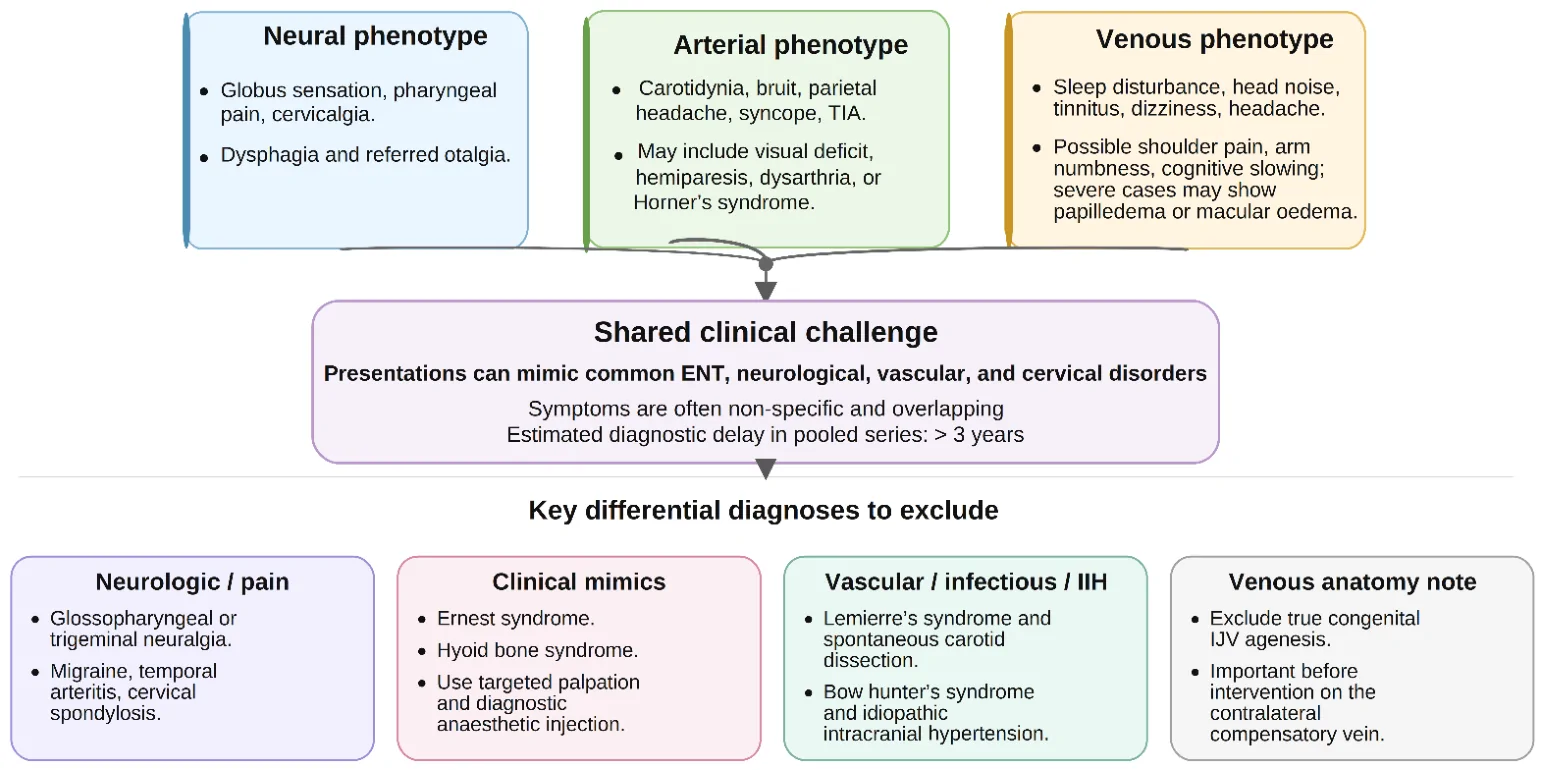
Phenotypic presentations of stylohyoid complex compression syndromes and diagnostic work-up overview. Upper panel: the three core clinical phenotypes—neural (globus sensation, pharyngeal pain, cervicalgia, dysphagia, referred otalgia), arterial (carotidynia, parietal headache, TIA, Horner’s syndrome, hemiparesis, dysarthria), and venous (sleep disturbance, tinnitus, dizziness, headache; papilledema or macular oedema in severe cases)—converge on a shared diagnostic challenge: symptoms frequently mimic ENT, neurological, vascular, and cervical disorders, and pooled case series report an estimated diagnostic delay exceeding three years. Lower panel: key differential diagnoses to exclude, grouped by neurological and pain mimics (glossopharyngeal and trigeminal neuralgia, migraine, temporal arteritis, cervical spondylosis), clinical mimics distinguishable by targeted palpation and diagnostic anaesthetic injection (Ernest syndrome, hyoid bone syndrome), vascular and infectious conditions (Lemièrre’s syndrome, spontaneous carotid dissection, bow hunter’s syndrome, idiopathic intracranial hypertension), and venous anatomical notes (congenital IJV agenesis, important to identify before any intervention on the contralateral compensatory vein). ENT, ear, nose and throat; TIA, transient ischaemic attack; IIH, idiopathic intracranial hypertension; IJV, internal jugular vein.

Differential diagnoses requiring exclusion (Figure 3) include glossopharyngeal and trigeminal neuralgia, migraine, temporal arteritis, and cervical spondylosis [4,6,8,12]. Ernest syndrome (stylomandibular ligament tendinosis) and hyoid bone syndrome are clinically distinguishable mimics, each confirmed by targeted palpation and diagnostic anaesthetic injection rather than imaging alone [4,9,16]. Lemierre’s syndrome, spontaneous carotid dissection due to connective tissue disease, vertebral artery bow hunter’s syndrome, and primary idiopathic intracranial hypertension should also be considered [6,12,22,39,62]. True congenital IJV agenesis is rare but clinically important to identify before any intervention on the contralateral, compensatory vein [16].

## 6. Management Strategies

### 6.1. Conservative Treatment

Management options by phenotype, spanning first-line and escalation strategies, are summarised in Table 3. Conservative management is generally regarded as first-line for mild to moderate symptoms or for patients who are not surgical candidates [2,4,6,10,71]. Local infiltration with lidocaine and corticosteroid into the tonsillar fossa provides temporary relief and diagnostic confirmation in the neural phenotype [4,5,7,10], and oral neuropathic agents are used for persistent neural pain. Where the presentation is indistinguishable from classic glossopharyngeal neuralgia, carbamazepine or oxcarbazepine is the accepted first-line agent, with gabapentin as an alternative in patients intolerant of them [74]. Pharmacological response is not, however, discriminating: relief with an anticonvulsant does not distinguish styloid-related neural compression from idiopathic neuralgia, and imaging remains necessary to identify a mechanical cause amenable to decompression [4,5,6]. For acute carotid dissection, antiplatelet or anticoagulant therapy is used to reduce thromboembolic risk. However, recurrence after medical management alone has been reported in approximately one-third of styloid-related dissection cases in one series, reflecting the persistence of the underlying mechanical conflict if left untreated [71]. For muscular venous entrapment, a structured physiotherapy programme of at least two months is generally recommended as the initial approach, with ultrasound-guided botulinum toxin type A reserved for non-responders in case-series data [22].

### 6.2. Surgical and Endovascular Treatment

Surgical SP resection (styloidectomy) is widely regarded as the definitive treatment for severe or persistent mechanical compression [4,5,6,71]. A meta-analysis comparing surgical and medical management reported substantially higher rates of symptom improvement or resolution with surgery (97.8%) than with medical treatment alone (65.9%) [6]; this is a pooled retrospective comparison rather than a randomised trial, and selection bias toward surgery in more severe or refractory cases cannot be excluded. The transoral approach offers a shorter operative time and no external scarring but more limited vascular control. In contrast, the transcervical approach is generally preferred for vascular and venous phenotypes because it affords wider exposure and the option of concurrent C1 tuberculectomy, with a low reported rate of transient marginal mandibular nerve weakness in case-series data [4,5,6,71]. For SJVCS, resection of the C1 lateral mass and styloid process—performed with concurrent balloon dilation—has been reported to increase J3 diameter with a fall in flow velocity in an individual case [17], and similarly favourable individual outcomes have been reported for combined arterial-venous decompression in mixed-phenotype cases [54] and for surgical release in jugular bow hunter’s syndrome [23]; these remain case-level observations rather than comparative trial data.

Endovascular IJV stenting is generally reserved for focal stenosis with a persistent trans-stenotic pressure gradient after conservative management. It is considered contraindicated as primary therapy where rigid external bony compression is unaddressed, since the stent itself remains vulnerable to crushing or migration under ongoing compression [2,18,27]. In the largest available case series of IJV stenting (33 procedures in 29 patients), periprocedural complications occurred in approximately one-third of procedures, including spinal accessory nerve palsy and, in one case, intracardiac stent migration; durable improvement at follow-up was reported in just over one-third of patients [27]. Based on this complication profile, a “styloidectomy-first” approach—surgical decompression first, with endovascular stenting reserved as a secondary or rescue option for persistent stenosis—is proposed on the basis of single-centre observational series, with no randomised comparison available, by several authors as an emerging preference. However, this remains the authors’ and others’ proposed pathway pending prospective comparative evidence rather than a universally accepted standard of care [27]. Biomechanical support for a decompression-first strategy comes from a 3D-printed craniocervical model of styloidogenic jugular compression, in which simulated intracranial pressure rose in proportion to ipsilateral axial rotation and IJV compression was relieved once approximately 75% of the styloid was resected, indicating that partial or complete styloidectomy can mechanically abolish the positional obstruction [75]. For acute carotid dissection, endovascular stabilisation followed by elective styloidectomy once stable is reported to reduce the risk of long-term stent deformation and recurrent dissection in case-series data [12,71].

## 7. Controversies, Terminology, and Future Directions

Whether the venous phenotype (SJVCS) should remain within the Eagle syndrome spectrum or be classified as a distinct entity is debated [15,16,18,76]. Proponents of unified terminology cite shared anatomical drivers and the practical value of neurosurgical awareness during posterior fossa surgery [15,58]. In contrast, others argue that the haemodynamics, presentation, and treatment of SJVCS are sufficiently distinct to warrant separate classification, and that the eponym “Eagle jugular syndrome” should be abandoned in favour of anatomically precise terminology [16,18,76]. Notably, the potential severity of the venous phenotype has arguably been under-emphasised in earlier, arterially centred accounts of the syndrome: styloidogenic jugular compression has been linked to cerebral venous sinus thrombosis and framed as a potentially life-threatening rather than merely positional disorder, which further supports treating it as a clinically distinct entity rather than a minor variant [20,77]. Throughout this review, “styloidogenic jugular venous compression syndrome” has been used as the preferred term; readers should be aware that the Styloid Jugular Nutcracker, Eagle jugular syndrome, jugular nutcracker syndrome, and JEDI syndrome appear in the literature as alternative or historical labels for overlapping or related entities [11,18,22,43,64,76]. The related term chronic cerebrospinal venous insufficiency (CCSVI) is used cautiously here and only where the cited source explicitly used it; the multiple sclerosis venous hypothesis with which CCSVI became closely associated has since been substantially discredited by multiple sham-controlled trials and a Cochrane systematic review that found no evidence that CCSVI causes MS or that venous angioplasty modifies its course. Its use here refers solely to the venous anatomical phenotype described in the cited source and should not be read as endorsing that broader hypothesis. de Bakker et al. (2019) have proposed “second pharyngeal arch cartilage anomalies” as a unifying embryological term for this group of conditions [76].

Western literature has more frequently associated IJVS with Ehlers–Danlos syndrome and multiple sclerosis [27]. In contrast, a large Chinese cohort reported no cases of multiple sclerosis and instead identified previous HBV infection and type 2 diabetes mellitus as the dominant systemic associations [61]. Both observations are drawn from single, geographically specific cohorts, and whether they reflect true population differences, referral bias, or differences in case ascertainment cannot be determined from the available data; this is presented as an open question rather than an established epidemiological contrast. Future classification work would benefit from prospectively defined cohorts, explicit separation of compressive from non-compressive IJVS, and pre-specified criteria correlating anatomical narrowing with haemodynamic and clinical findings before invasive decompression is recommended [3,21,72]. Existing classifications have largely stratified the styloid process by length alone (for example the tripartite radiographic scheme of Sokler 2001), an approach that predates recognition of the venous phenotype and does not capture three-dimensional orientation, vessel relationship, or the neural, arterial and venous mechanisms distinguished here; the framework proposed in this review is intended to address that gap rather than to refine a length threshold [78].

Three practical difficulties should be anticipated if the framework proposed here is applied prospectively. First, the separation of subtypes IIa and IIb depends on demonstrating arterial wall injury, which requires cross-sectional or catheter imaging that is often unavailable at the point of first presentation; where a patient presents acutely with movement-provoked neurological symptoms, the safe default is to manage as IIb until dissection has been excluded, rather than to attempt subtype assignment on clinical grounds. Second, no validated threshold separates the dynamic and static venous subtypes. The distinction as used here rests on whether stenosis persists in the neutral position, which is a working convention rather than a criterion, and cases with intermediate behaviour—partial reduction on return to neutral—are not accommodated by a binary division. Third, because phenotypes overlap and may progress, assignment describes a patient at a point in time rather than a fixed category, and reassignment should be expected during follow-up. These limitations are inherent to a framework that has not yet been tested against outcome data, and they define the questions that prospective evaluation would need to address.

## 8. Limitations of the Current Evidence Base

Several limitations should be borne in mind when applying the findings summarised in this review. First, this is a narrative rather than systematic review; sources were not identified through a pre-registered search strategy, and publication bias toward unusual or successfully treated cases cannot be excluded, particularly for the venous phenotype. Second, much of the venous-phenotype literature derives from small, single-centre retrospective series or individual case reports, and prevalence figures drawn from these sources describe the reported case mix rather than population incidence. Third, terminology for the venous phenotype remains unsettled across the source literature, which limits direct comparison between studies using different diagnostic thresholds or labels. Fourth, the proposed four-tier classification and the “styloidectomy-first” management algorithm presented here are syntheses proposed by the authors rather than externally validated frameworks, and should be treated as a starting point for prospective evaluation rather than as established clinical standards. Finally, several systemic associations discussed (HBV infection, type 2 diabetes mellitus, connective tissue disorders) are drawn from single cohorts and require independent replication before they can be considered generalisable risk factors. It should also be acknowledged that the evidence base for the vascular phenotype, while still dominated by case reports, now includes a formal systematic review of Eagle-related vascular complications [79]; several earlier narrative reviews and single-case reports drew relatively firm mechanistic conclusions from limited data, and their inferences are better read in light of this higher-tier synthesis.

The evidence-quality tiers applied throughout this review, from systematic-review-level synthesis to speculative, unvalidated mechanistic proposals, are summarised in Table 4.

## 9. Conclusions

Compression syndromes of the stylohyoid complex span neural, arterial, and venous phenotypes that share a common anatomical corridor but differ substantially in mechanism, presentation, and evidentiary support. SP elongation is a frequent anatomical variant, but the available evidence consistently indicates that length alone is an insufficient predictor of symptomatic compression; three-dimensional orientation, proximity to adjacent vessels, and dynamic musculoskeletal interactions appear more relevant, though largely based on retrospective and cross-sectional data. Dynamic vascular imaging in provocative head positions adds meaningful diagnostic information beyond static anatomical assessment, particularly for the venous phenotype. Still, anatomical narrowing must be interpreted alongside clinical and haemodynamic correlation given its high incidental prevalence. Surgical decompression appears favourable relative to conservative management in the retrospective literature available to date, and a “styloidectomy-first” approach is a reasonable proposed strategy for osseous venous compression pending prospective validation; primary endovascular stenting carries a non-trivial complication profile and is best reserved for refractory or combined presentations. A standardised classification and terminology, of the kind proposed here, may help future studies define these phenotypes consistently. Still, such a framework will require prospective testing before it can be considered established practice.

## Figures and Tables

**Table 1:** Proposed classification of stylohyoid-complex compression phenotypes. 3D, three-dimensional; CN, cranial nerve; CT, computed tomography; CTA, computed tomography angiography; CTV, computed tomography venography; ECA, external carotid artery; ICA, internal carotid artery; IJV, internal jugular vein; OPG, orthopantomogram; SCM, sternocleidomastoid; SP, styloid process; TIA, transient ischaemic attack.

Type	Primary Structure Compressed	Mechanism	Typical Symptoms	Key Imaging	Main Treatment
I—Neural	CN IX (± V, VII, X)	Scar/ligament tethering nerve over SP tip	Pharyngeal pain, otalgia, globus, dysphagia	3D-CT; OPG screening	Carbamazepine/oxcarbazepine; local anaesthetic block; styloidectomy if refractory
IIa—Arterial (mid-ICA)	Internal carotid artery	SP impingement on adventitia/plexus	Caroticodynia, parietal headache, TIA	3D-CTA	Styloidectomy ± endovascular stabilisation
IIb—Arterial (proximal/mid-ICA)	Internal carotid artery	As IIa, higher thromboembolic risk	TIA, stroke risk	3D-CTA; catheter angiography	Anticoagulation; staged styloidectomy
IIc—Arterial (ECA)	External carotid artery	Retrostyloid ECA impingement	Facial/orbital pain	3D-CTA	Styloidectomy
IIIa—Venous (dynamic)	IJV (J3)	Rotational SP/C1 or SCM entrapment	Positional headache, tinnitus, vertigo	Dynamic CTV/venography	Physiotherapy; styloidectomy if persistent
IIIb—Venous (static)	IJV (J3)	Fixed SP–C1 bony narrowing	Chronic headache, pulsatile tinnitus, papilledema (selected cases)	3D-CTV; catheter manometry	“Styloidectomy-first” (proposed); stenting if refractory
IV—Mixed	Combined arterial + venous	Concurrent compression of multiple structures	Combined arterial and venous features	Multimodal (3D-CTA/CTV)	Individualised staged surgery

**Table 2:** Diagnostic modalities and their principal contribution. 3D, three-dimensional; CT, computed tomography; CTA, computed tomography angiography; CTV, computed tomography venography; ICA, internal carotid artery; MRA, magnetic resonance angiography; OPG, orthopantomogram; SP, styloid process.

Modality	Primary Role	Key Limitation
Plain radiography/OPG	Initial screening for SP length and calcification; suitable for first-line diagnosis and epidemiological survey given low dose and cost [73].	Two-dimensional projection: cannot assess three-dimensional angulation, deviation, or relationship to adjacent vessels; susceptible to distortion, magnification, and overlap of surrounding structures. Length measurement itself correlates closely with CT (r = 0.92–0.97) [36].
3D-CTA/CTV	Anatomical cornerstone: SP morphology, vessel proximity, dynamic positional imaging.	Incidental stenosis common in asymptomatic individuals.
Quantitative Doppler ultrasound	Real-time flow velocity and positional change.	Operator-dependent; limited deep-corridor visualisation.
Magnetic resonance venography/MRA/black-blood imaging	Stenosis localisation; carotid dissection; dural sinus patency.	Less precise bony detail than CT.
Catheter venography with manometry	Haemodynamic reference standard; trans-stenotic gradient.	Invasive; reserved for selected symptomatic cases.
Transoral carotid ultrasonography	Real-time assessment of the extracranial ICA lumen; detects dissection (double-lumen flow); arterial phenotype (Types IIa–b) [51].	Limited availability; operator-dependent; restricted to carotid assessment; cannot visualise bony conflict geometry.

**Table 3:** Management options by phenotype. BoNT-A, botulinum toxin type A; NSAID, non-steroidal anti-inflammatory drug; SJVCS, styloidogenic jugular venous compression syndrome.

Phenotype	First-Line	Escalation	Evidence Basis
Neural	Anaesthetic/steroid infiltration; NSAIDs; neuropathic agents	Transoral or transcervical styloidectomy	Retrospective case series; one large surgical-vs-medical meta-analysis.
Arterial	Antiplatelet/anticoagulant if dissection	Endovascular stabilisation, then elective styloidectomy	Pooled case reports; single-centre series.
Venous (dynamic)	Physiotherapy ± BoNT-A for muscular entrapment	Surgical release (myotomy) if refractory	Small case series.
Venous (static, SJVCS)	Conservative trial where mild	“Styloidectomy-first” (proposed); secondary stenting if refractory	Retrospective series; no randomised data.
Mixed	Individualised, staged	Combined arterial and venous surgical decompression	Individual case reports.

**Table 4:** Evidence-quality grading applied in this review. HBV, hepatitis B virus.

Evidence Tier	Examples in This Review	Interpretive Framing Used
Stronger (systematic review/meta-analysis)	[13] (styloid prevalence); [6] (surgical vs. medical outcomes)	“Reported prevalence,” “pooled estimate”
Moderate (defined observational cohort/series, prospective or retrospective)	[17,21,27,40,61]	“In selected symptomatic cohorts,” “in one series”
Weak/emerging (small series or case reports)	[15,23,24,62]	“Has been described,” “may occur”
Speculative (proposed mechanism, unvalidated)	HBV/immunopathic vasculitis pathway [61]; proposed four-tier classification; “styloidectomy-first” algorithm	“Hypothesised,” “proposed,” “requires validation”

## Data Availability

No new data were generated or analysed in support of this review. All data discussed are available in the cited published sources.

## Abbreviations

The following abbreviations are used in this manuscript:

3D	three-dimensional
BoNT-A	botulinum toxin type A
C1	first cervical vertebra
C2	second cervical vertebra
CCSVI	chronic cerebrospinal venous insufficiency
CN	cranial nerve
CT	computed tomography
CTA	computed tomography angiography
CTV	computed tomography venography
ECA	external carotid artery
ESP	elongated styloid process
HBV	hepatitis B virus
ICA	internal carotid artery
IJV	internal jugular vein
J3	third (jugular foramen) segment of the internal jugular vein
MRA	magnetic resonance angiography
NSAID	non-steroidal anti-inflammatory drug
OPG	orthopantomogram
SCM	sternocleidomastoid
SHC	stylohyoid complex
SHL	stylohyoid ligament
SJVCS	styloidogenic jugular venous compression syndrome
SP	styloid process
TIA	transient ischaemic attack

## References

[1] Eagle W.W. (1948). Elongated styloid process; further observations and a new syndrome. Arch. Otolaryngol..

[2] Li M., Su C., Fan C., Chan C.C., Bai C., Meng R. (2019). Internal jugular vein stenosis induced by tortuous internal carotid artery compression: Two case reports and literature review. J. Int. Med. Res..

[3] Mantovani G., Zangrossi P., Flacco M.E., Di Domenico G., Nastro Siniscalchi E., De Ponte F.S., Maugeri R., De Bonis P., Cavallo M.A., Zamboni P. (2023). Styloid Jugular Nutcracker: The Possible Role of the Styloid Process Spatial Orientation-A Preliminary Morphometric Computed Study. Diagnostics.

[4] Piagkou M., Anagnostopoulou S., Kouladouros K., Piagkos G. (2009). Eagle’s syndrome: A review of the literature. Clin. Anat..

[5] Shahoon H., Kianbakht C. (2008). Symptomatic Elongated Styloid Process or Eagle’s Syndrome: A Case Report. J. Dent. Res. Dent. Clin. Dent. Prospect..

[6] Hassani M., Gronlund E.W., Albrechtsen S.S., Kondziella D. (2024). Neurological phenotypes and treatment outcomes in Eagle syndrome: Systematic review and meta-analysis. PeerJ.

[7] Costantinides F., Vidoni G., Bodin C., Di Lenarda R. (2013). Eagle’s syndrome: Signs and symptoms. Cranio.

[8] Eagle W.W. (1958). Elongated styloid process; symptoms and treatment. AMA Arch. Otolaryngol..

[9] Shankland W.E. (2010). Anterior throat pain syndromes: Causes for undiagnosed craniofacial pain. Cranio.

[10] Sharma N., Ram R., Kamal R. (2016). Unusually elongated styloid process: A report of two cases with literature review. Ann. Maxillofac. Surg..

[11] Zamboni P., Scerrati A., Menegatti E., Galeotti R., Lapparelli M., Traina L., Tessari M., Ciorba A., De Bonis P., Pelucchi S. (2019). The eagle jugular syndrome. BMC Neurol..

[12] Pagano S., Ricciuti V., Mancini F., Barbieri F.R., Chegai F., Marini A., Marruzzo D., Paracino R., Ricciuti R.A. (2023). Eagle syndrome: An updated review. Surg. Neurol. Int..

[13] Triantafyllou G., Paschopoulos I., Duparc F., Tsakotos G., Papadopoulos-Manolarakis P., Piagkou M. (2025). The Anatomy of the Stylohyoid Chain: A Systematic Review with Meta-Analysis. Diagnostics.

[14] Badhey A., Jategaonkar A., Kovacs A.J.A., Kadakia S., De Deyn P.P., Ducic Y., Schantz S., Shin E. (2017). Eagle syndrome: A comprehensive review. Clin. Neurol. Neurosurg..

[15] Nonaka T., Sakata K., Abe T., Hattori G., Orito K., Miyagi N., Tokutomi T., Morioka M. (2021). The eagle jugular syndrome as the cause of delayed intracranial hemorrhage after microvascular decompression for hemifacial spasm: A case report. Surg. Neurol. Int..

[16] Dinca V., Ionescu P., Tudose R.C., Munteanu M., Vrapciu A.D., Rusu M.C. (2025). Anatomical Reasons for an Impaired Internal Jugular Flow. Medicina.

[17] Ding J.Y., Zhou D., Pan L.Q., Ya J.Y., Liu C., Yan F., Fan C.Q., Ding Y.C., Ji X.M., Meng R. (2020). Cervical spondylotic internal jugular venous compression syndrome. CNS Neurosci. Ther..

[18] Angelini C., Mantovani G., Cavallo M.A., Scerrati A., De Bonis P. (2023). Neurosurgical implications of the Jugular Vein Nutcracker. Veins Lymphat..

[19] Gianesini S., Menegatti E., Mascoli F., Salvi F., Bastianello S., Zamboni P. (2014). The omohyoid muscle entrapment of the internal jugular vein. A still unclear pathogenetic mechanism. Phlebology.

[20] Guan J., Song S., Wang W., Ji X., Meng R. (2021). Cerebral venous sinus thrombosis due to external compression of internal jugular vein. J. Int. Med. Res..

[21] Scerrati A., Norri N., Mongardi L., Dones F., Ricciardi L., Trevisi G., Menegatti E., Zamboni P., Cavallo M.A., De Bonis P. (2021). Styloidogenic-cervical spondylotic internal jugular venous compression, a vascular disease related to several clinical neurological manifestations: Diagnosis and treatment-a comprehensive literature review. Ann. Transl. Med..

[22] Cha Y.H., Gharib M., Chan K., Karam J. (2025). Sternocleidomastoid Omohyoid Entrapment of the Internal Jugular Vein Causing Vertigo and Headaches. Clin. Otolaryngol..

[23] Brinjikji W., Graffeo C.S., Perry A., Zimmerman T., Janus J.R., Morris P.P., Cascino G.D., Lanzino G. (2017). Moving target: Transient rotational stenosis precipitating jugular bow hunter’s syndrome. J. Neurointerv. Surg..

[24] Ichiyama S., Nomura O., Ishizawa Y. (2023). Disruption of the internal jugular vein by subcutaneous emphysema. BMJ Case Rep..

[25] Baethge C., Goldbeck-Wood S., Mertens S. (2019). SANRA-a scale for the quality assessment of narrative review articles. Res. Integr. Peer Rev..

[26] Natsis K., Repousi E., Noussios G., Papathanasiou E., Apostolidis S., Piagkou M. (2015). The styloid process in a Greek population: An anatomical study with clinical implications. Anat. Sci. Int..

[27] Fargen K.M., Midtlien J.P., Belanger K., Hepworth E.J., Hui F.K. (2024). The Promise, Mystery, and Perils of Stenting for Symptomatic Internal Jugular Vein Stenosis: A Case Series. Neurosurgery.

[28] Calota R.N., Rusu M.C., Vrapciu A.D. (2024). The External Carotid Artery and the Styloid Process. Curr. Health Sci. J..

[29] Li L., Li P., London N.R., Xu H., Chen X., Carrau R.L. (2023). Relevance of the Internal Jugular Vein for Surgery in the Upper Parapharyngeal Space. Ear Nose Throat J..

[30] Triantafyllou G., Botis G., Vassiou K., Vlychou M., Tsakotos G., Kalamatianos T., Matsopoulos G., Piagkou M. (2025). The styloid process length and the stylohyoid chain ossification affect its relationship with the carotid arteries. Ann. Anat..

[31] Rusu M.C., Zamfirescu V.I., Tudose R.C. (2026). Distinguishing true from pseudo-absent styloid process: A case-prompted critical analysis. Surg. Radiol. Anat..

[32] Balcioglu H.A., Kilic C., Akyol M., Ozan H., Kokten G. (2009). Length of the styloid process and anatomical implications for Eagle’s syndrome. Folia Morphol..

[33] Guarna M., Lorenzoni P., Volpi N., Aglianò M. (2021). Elongated styloid process: Literature review and morphometric data on a collection of dried skulls. Ital. J. Anat. Embryol..

[34] Ekici F., Tekbas G., Hamidi C., Onder H., Goya C., Cetincakmak M.G., Gumus H., Uyar A., Bilici A. (2013). The distribution of stylohyoid chain anatomic variations by age groups and gender: An analysis using MDCT. Eur. Arch. Oto-Rhino-Laryngol..

[35] Assiri Ahmed H., Estrugo-Devesa A., Rosello Llabres X., Egido-Moreno S., Lopez-Lopez J. (2023). The prevalence of elongated styloid process in the population of Barcelona: A cross-sectional study & review of literature. BMC Oral Health.

[36] Dudde F., Bergmann W., Schuck O., Schunk J., Barbarewicz F. (2024). Accuracy in the Diagnostics of Styloid Process—Panoramic Radiograph vs. Computed Tomography: A Comparative Study. In Vivo.

[37] Steinman E.P. (1968). Styloid Syndrome in Absence of an Elongated Process. Acta Oto-Laryngol..

[38] Kent D.T., Rath T.J., Snyderman C. (2015). Conventional and 3-Dimensional Computerized Tomography in Eagle’s Syndrome, Glossopharyngeal Neuralgia, and Asymptomatic Controls. Otolaryngol. Head Neck Surg..

[39] Paulsen F., Tillmann B., Christofides C., Richter W., Koebke J. (2000). Curving and looping of the internal carotid artery in relation to the pharynx: Frequency, embryology and clinical implications. J. Anat..

[40] Fargen K.M., Midtlien J.P., Margraf C., Kiritsis N.R., Chang E., Hui F. (2025). Dynamic internal jugular vein venography: A descriptive study in 89 patients with suspected cerebral venous outflow disorders. J. Neurointerv. Surg..

[41] Jeyaraj P. (2022). Histopathological Analysis of Elongated Styloid Processes: A New Light on Etiopathogenesis of Eagle’s Syndrome. Indian J. Otolaryngol. Head Neck Surg..

[42] Kim S.M., Seo M.H., Myoung H., Choi J.Y., Kim Y.S., Lee S.K. (2014). Osteogenetic changes in elongated styloid processes of Eagle syndrome patients. J. Craniomaxillofac. Surg..

[43] Dilek F., Cosgunarslan A., Canger E.M. (2023). Evaluation of alterations in length and calcification of the styloid process in patients with end-stage renal failure. Oral Surg. Oral Med. Oral Pathol. Oral Radiol..

[44] Gokce C., Sisman Y., Sipahioglu M. (2008). Styloid Process Elongation or Eagle’s Syndrome: Is There Any Role for Ectopic Calcification?. Eur. J. Dent..

[45] Sisman Y., Gokce C., Sipahioglu M. (2009). Bilateral Elongated Styloid Process in an End-stage Renal Disease Patient with Peritoneal Dialysis: Is there Any Role for Ectopic Calcification?. Eur. J. Dent..

[46] Caraballo-Meza S., Barakat-Polo N., Plazas-Roman J., Diaz-Caballero A., Ardila C.M. (2025). Predictive Models for Stylohyoid Complex Elongation: A Multivariate Statistical Analysis with Evidence-Based Diagnostic Criteria. J. Clin. Exp. Dent..

[47] Gooris P.J., Zijlmans J.C., Bergsma J.E., Mensink G. (2014). A case of mental nerve paresthesia due to dynamic compression of alveolar inferior nerve along an elongated styloid process. J. Oral Maxillofac. Surg..

[48] Hoffmann E., Rader C., Fuhrmann H., Maurer P. (2013). Styloid-carotid artery syndrome treated surgically with Piezosurgery: A case report and literature review. J. Craniomaxillofac. Surg..

[49] Raser J.M., Mullen M.T., Kasner S.E., Cucchiara B.L., Messe S.R. (2011). Cervical carotid artery dissection is associated with styloid process length. Neurology.

[50] Razak A., Short J.L., Hussain S.I. (2014). Carotid artery dissection due to elongated styloid process: A self-stabbing phenomenon. J. Neuroimaging.

[51] Ohara N., Sakaguchi M., Okazaki S., Nagano K., Kitagawa K. (2012). Internal carotid artery dissection caused by an elongated styloid process: Usefulness of transoral ultrasonography. J. Stroke Cerebrovasc. Dis..

[52] Chuang W.C., Short J.H., McKinney A.M., Anker L., Knoll B., McKinney Z.J. (2007). Reversible left hemispheric ischemia secondary to carotid compression in Eagle syndrome: Surgical and CT angiographic correlation. AJNR Am. J. Neuroradiol..

[53] Tadjer J., Béjot Y. (2024). Vascular variant of Eagle syndrome: A review. Front Neurol..

[54] Sultan S., Acharya Y., Soliman O., Hynes N. (2022). Stylohyoid Eagle syndrome and EXTracranial INternal Carotid arTery pseudoaneurysms (EXTINCT) with internal jugular vein nutcracker syndrome: A challenging clinical scenario. BMJ Case Rep..

[55] Shchanitsyn I.N., Shumilina M.V., Arakelyan V.S., Perepechin E.A., Nikiforova M.A. (2024). Shilopodilingual syndrome as a cause of carotid artery dissection (literature review). Angiol. Sosud. Khir..

[56] Abdelnour L.H., Kurdy M., Idris A. (2023). Association of styloid process length with cervical carotid artery dissection: Meta-analysis. Health Sci. Rev..

[57] Lahlali K., Karimi J., Ouazzani H., Chaouche I., Akammar A., El Bouardi N., Bouchal S., Chtaou N., Alami B., Boubbou M. (2026). Mechanical stress from the hyoid bone as a potential etiology of bony-related ischemic stroke secondary to carotid artery dissection: A case report and systematic review. Radiol. Case Rep..

[58] Seoane E., Rhoton A.L. (1999). Compression of the internal jugular vein by the transverse process of the atlas as the cause of cerebellar hemorrhage after supratentorial craniotomy. Surg. Neurol..

[59] Motoyama Y., Sasaki H., Nakajima T., Hayami H., Matsuoka R., Fukutome K., Tei R., Shin Y., Aketa S. (2024). Eagle jugular syndrome accompanied by de novo brainstem cavernous malformation: A case-based systematic review. Acta Neurochir..

[60] Pereira F.L., Filho L.I., Pavan A.J., Farah G.J., Goncalves E.A., Veltrini V.C., Camarini E.T. (2007). Styloid-stylohyoid syndrome: Literature review and case report. J. Oral Maxillofac. Surg..

[61] Bai C., Ding J., Da Z., Sun J., Liu C., Pan L., Ya J., Wang Z., Guan J., Jin K. (2020). Probable risk factors of internal jugular vein stenosis in Chinese patients-A real-world cohort study. Clin. Neurol. Neurosurg..

[62] Han A.Y., Lentz J.F., Kuan E.C., Araya H.H., Kamgar M. (2016). A Case of Reactive Cervical Lymphadenopathy with Fat Necrosis Impinging on Adjacent Vascular Structures. Case Rep. Otolaryngol..

[63] Oushy S., Wald J.T., Janus J., Fulgham J.R., Lanzino G. (2020). Dynamic Internal Jugular Vein Compression by Hypertrophic Hyoid Bone: Management and Outcomes. Cureus.

[64] Simka M., Majewski E., Fortuna M., Zaniewski M. (2012). Internal jugular vein entrapment in a multiple sclerosis patient. Case Rep. Surg..

[65] Gailloud P., Fasel J.H., Muster M., Desarzens F., Ruefenacht D.A. (1997). Termination of the inferior petrosal sinus: An anatomical variant. Clin. Anat..

[66] Zhang W.G., Ye Y.Y., Chen J.H., Chen R., Kuang L.Q., Li X. (2010). Imaging study of the long extracranial extension of the inferior petrosal sinus with MSCT. Clin. Anat..

[67] Hartl D.M., Zafereo M.E., Kowalski L.P., Randolph G.W., Olsen K.D., Fernandez-Alvarez V., Nixon I.J., Shaha A.R., Angelos P., Shah J.P. (2021). Occlusion of the internal jugular vein in differentiated thyroid carcinoma: Causes and diagnosis. Eur. J. Surg. Oncol..

[68] Das K.K., Mehrotra A., Sahu R.N., Srivastava A.K., Jaiswal A.K., Behari S. (2013). Unilateral lateral mass hypertrophy: An extremely rare congenital anomaly of atlas. J. Craniovertebr. Junction Spine.

[69] Onishi E., Sakamoto A., Murata S., Nakamura S., Matsushita M. (2013). Unilateral atlantal lateral mass hypertrophy associated with atlanto-occipital fusion. Eur. Spine J..

[70] Sekerci A.E., Soylu E., Arikan M.P., Aglarci O.S. (2015). Is there a relationship between the presence of ponticulus posticus and elongated styloid process?. Clin. Imaging.

[71] Klail T., Sachs L., Panos L.D., Urban O.Y., Siller T., Pilgram-Pastor S., Giger R., Muller M., Wagner F. (2025). Styloid process-related internal carotid artery dissection: Extensive literature review of diagnosis, treatment and outcomes (exemplified with a single-center case series). Neuroradiology.

[72] Jayaraman M.V., Boxerman J.L., Davis L.M., Haas R.A., Rogg J.M. (2012). Incidence of extrinsic compression of the internal jugular vein in unselected patients undergoing CT angiography. AJNR Am. J. Neuroradiol..

[73] Bruno G., De Stefani A., Barone M., Costa G., Saccomanno S., Gracco A. (2022). The validity of panoramic radiograph as a diagnostic method for elongated styloid process: A systematic review. Cranio.

[74] Han A., Montgomery C., Zamora A., Winder E., Kaye A., Carroll C., Aquino A., Kakazu J., Kaye A.D. (2022). Glossopharyngeal Neuralgia: Epidemiology, Risk factors, Pathophysiology, Differential diagnosis, and Treatment Options. Health Psychol. Res..

[75] Lehrman J.N., Narayanan M., Cavallo C., Newcomb A., Zhao X., Kelly B.P., Crawford N.R., Nakaji P. (2020). Evaluation of abnormal styloid anatomy as a cause of internal jugular vein compression using a 3D-printed model: A laboratory investigation. J. Clin. Neurosci..

[76] de Bakker B.S., de Bakker H.M., Soerdjbalie-Maikoe V., Dikkers F.G. (2019). Variants of the hyoid-larynx complex, with implications for forensic science and consequence for the diagnosis of Eagle’s syndrome. Sci. Rep..

[77] Siniscalchi E.N., Raffa G., Germanò A., De Ponte F.S. (2020). Is eagle jugular syndrome an underestimated potentially life-threatening disease?. Can. J. Neurol. Sci..

[78] Sokler K., Sandev S. (2001). New classification of the styloid process length--clinical application on the biological base. Coll. Antropol..

[79] Thielen A., Brizzi V., Majoufre C., Nicot R., Schlund M. (2025). Eagle syndrome and vascular complications-a systematic review. Int. J. Oral Maxillofac. Surg..

